# AquaYOLO: Advanced YOLO-based fish detection for optimized aquaculture pond monitoring

**DOI:** 10.1038/s41598-025-89611-y

**Published:** 2025-02-20

**Authors:** M. Vijayalakshmi, A. Sasithradevi

**Affiliations:** 1 School of Electronics Engineering, Vellore Institute of Technology, Chennai, 600127 India; 2 Center for Advanced Data Science, Vellore Institute of Technology, Chennai, 600127 India

**Keywords:** Fish Detection, Hierarchical features, Aquaculture Monitoring, Deep Learning, YOLO, Data acquisition, Image processing, Ecology, Ecology

## Abstract

Aquaculture plays an important role in ensuring global food security, supporting economic growth, and protecting natural resources. However, traditional methods of monitoring aquatic environments are time-consuming and labor-intensive. To address this, there is growing interest in using computer vision for more efficient aqua monitoring. Fish detection is a key challenging step in these vision-based systems, as it faces challenges such as changing light conditions, varying water clarity, different types of vegetation, and dynamic backgrounds. To overcome these challenges, we introduce a new model called AquaYOLO, an optimized model specifically designed for aquaculture applications. The backbone of AquaYOLO employs CSP layers and enhanced convolutional operations to extract hierarchical features. The head enhances feature representation through upsampling, concatenation, and multi-scale fusion. The detection head uses a precise 40 × 40 scale for box regression and dropping the final C2f layer to ensure accurate localization. To test the AquaYOLO model, we utilize DePondFi dataset (Detection of Pond Fish) collected from aquaponds in South India. DePondFi dataset contains around 50k bounding box annotations across 8150 images. Proposed AquaYOLO model performs well, achieving a precision, recall and mAP@50 of 0.889, 0.848, and 0.909 respectively. Our model ensures efficient and affordable fish detection for small-scale aquaculture.

## Introduction

India’s cultural and economic landscape is shaped by its diverse fish farming practices, which play a crucial role in supporting the economy, ensuring food security, and maintaining ecological balance. As the Indian economy grows and seafood demand increases, fish farming has transitioned into a dynamic commercial industry. Pond fish culture, in particular, provides a stable source of protein-rich food, creates jobs in rural and coastal areas, and enhances environmental sustainability by improving water quality and supporting biodiversity. Traditional methods of fish pond monitoring are labor-intensive and inefficient, especially as aquaculture scales up. This highlights the need for innovative, automated systems to improve accuracy and streamline monitoring processes. Computer vision technologies^1^ provide an efficient solution by enabling real-time monitoring and management of aquaculture environments^2^. Fish detection is a primary and essential step in aquaculture monitoring. However, challenges such as varying lighting conditions, water turbidity, and aquatic vegetation significantly impact the accuracy of traditional monitoring techniques.

In recent years, deep learning techniques gained considerable interest for applications in detecting fish^3^. Deep learning techniques^4^ detect objects by automatically identifying unique features, unlike traditional methods that rely on manually crafted features. Object detection in deep learning is categorized into two approaches: one-stage approach and two-stage approach. One-stage object detection approaches, including YOLO^5^, SSD^6^, and RetinaNet^7^, integrate region proposal and object detection into a unified process. This design allows for faster processing and reduced complexity, making it highly suitable for real-time applications. Two-stage approaches, such as versions of R-CNN^8^, separate the region proposal generation and object detection tasks. While these approaches achieve higher accuracy, they require significantly more computational resources. EfficientNet^9^, commonly employed as a backbone network, can be integrated into both one-stage and two-stage approaches, enhancing feature extraction and improving model performance across a wide range of detection tasks. One-stage approaches are ideal for real-time applications in small-scale aquaculture due to their integrated detection pipeline, ensuring faster processing. The model efficiency and lower computational demands make them suitable for quick decision-making in dynamic aquatic environments^10^. YOLO’s^11^ adaptability to challenging environments and proven effectiveness in studies make it ideal for real-time applications in small-scale aquaculture. Additionally, its integration with lightweight architectures, such as MobileNet^12^, further enhances efficiency. Some studies have developed models like YOLO-Fish^13^ to tackle the complexities of identifying fish in underwater environments. YOLO-Fish-1 modifies YOLOv3 by adjusting the upsampling process to improve detection of small fish, while YOLO-Fish-2 adds Spatial Pyramid Pooling to handle dynamic and complex environments. Santoso et al.^14^ compared Faster R-CNN, SSD-MobileNet, and YOLOv5 for coral fish detection in aquatic environments, emphasizing their application in Autonomous Underwater Vehicles (AUVs). The results demonstrated the potential of these models in advancing underwater fish detection, with SSD-MobileNet being recommended for real-time applications due to its efficient balance of speed and accuracy. Cai et al. developed a NAM-YOLOv7^15^ hybrid model incorporating NAM attention and MPDIoU loss function to enhance feature precision and minimize detection errors. Mahoro et al.^16^ utilized YOLOv7 and DETR-ResNet-50 for fish detection and classification using high-resolution sonar data. The study highlighted YOLOv7’s superior performance with a mean average precision of 0.79, demonstrating its suitability for aquatic ecosystem monitoring. Yang et al. proposed UGC-YOLO^17^, integrating global semantics and multi-scale feature fusion to improve underwater object detection. It employed deformable convolutions for long-range dependencies and Pyramid Pooling Module (PPM) pooling for high-layer semantic aggregation. Shen et al.^18^ proposed a criss-cross global interaction strategy (CGIS) to improve underwater object detection by reducing background interference and enhancing object recognition. The CGIS framework incorporates feature decomposition and extraction through criss-cross structures for effective global information exchange. Zhang et al.^19^ introduced BG-YOLO, a bidirectional-guidance approach for detecting underwater objects in degraded conditions. It integrates parallel enhancement and detection branches, with a feature-guided module optimizing detection-relevant features. Zhang et al.^20^ introduced MAD-YOLO, an improved YOLOv5-based detection framework designed specifically for compact, small-scale marine benthic organisms in complex underwater conditions. The architecture incorporates VOVDarkNet as its backbone, utilizing intermediate features with diverse receptive fields to enhance adaptability. Recent advancements, such as AODEGRU^21^ and CGTFN^22^, have enhanced water quality analysis in aquaculture through hybrid architectures. AODEGRU utilizes attention mechanisms with GRU to analyze time-series data, improving the detection of water contamination. CGTFN integrates CNN with Gated Recurrent Units (GRU) and a Temporal Fusion mechanism to extract spatial and temporal features, providing robust water quality classification. Similarly, Fish-TViT^23^, a Vision Transformers, has shown exceptional performance in fish species classification across multi-water environments. These models, when combined with detection frameworks can enable comprehensive systems for smart aquaculture, addressing both water quality monitoring and real-time fish detection, thus enhancing efficiency and sustainability.

To effectively utilize these methodologies in real-time environments, the availability of large-scale datasets is crucial. The OzFish dataset^24^, developed under the Australian Research Data Commons Data Discoveries program. It comprises approximately 80,000 labeled fish crops from over 500 species and 45,000 bounding box annotations across 1,800 frames. The DeepFish dataset^25^ is a large-scale benchmark comprising approximately 40,000 underwater images from 20 tropical Australian habitats. Vijayalakshmi et al.^26^ curated Ich and EUS diseased fish dataset of 1000 images per category collected from Tamil Nadu aqua farms and internet sources. The LifeCLEF 2015 dataset^27^ consists of 20 videos manually annotated by two expert annotators, featuring 15 fish species with over 9000 annotations and more than 20,000 sample images, designed to familiarize researchers with fish identification methods. Md Shoaib Ahmed et al.^28^ created a novel dataset for salmon fresh and infected fish using images sourced from the internet and aquaculture firms, comprising 266 images and expanded to 1326 images after augmentation for effective disease identification. Ditria et al.^29^ used submerged action cameras to capture video recordings of luderick fish in the Tweed River Estuary, deploying six cameras for one hour across different seagrass patches, resulting in 6080 annotated images. Jager et al.^30^ used the SeaCLEF^31^ dataset to track fish in real-time, featuring images and videos of around 150 marine animal species globally, including twenty underwater videos from Taiwan’s coral reefs for automated fish detection and species recognition. Xu et al.^32^ analyzed fish behavior under varying ammonia concentrations using a custom dataset of 36,000 images sampled from a 10-hour video, with 1700 images annotated for training and testing. Monkman et al.^33^ used European sea bass images to estimate fish length, encompassing metadata like date, location, time, species information, and human activity data. Fish detection datasets, such as Fish4Knowledge^34^, WildFish database^35^, Labeled Fishes in the Wild, and Rockfish^36^, are essential for advancing species identification, monitoring behaviors, and supporting sustainable fisheries management. FishNet^37^ provided a large-scale dataset of 17,357 aquatic species, categorized by taxonomy, to support tasks like classification, detection, and functional trait prediction. It established a benchmark for advancing aquatic species recognition and facilitated research in aquatic ecology. By referring to the literature it is clear that there is no benchmark dataset publicly available for real-time monitoring in the South Indian Pond environment. In 2023, the aquaculture sector in South India continued to be a major economic contributor. India has approximately 2.36 million hectares of ponds and tanks dedicated to aquaculture, with a significant portion in South Indian states like Tamil Nadu, Andhra Pradesh^38^, and Kerala. If computer vision-based real-time monitoring is implemented, it could further enhance economic growth^39^. Also from the literature review, it is evident that YOLO is the most commonly used detection algorithm for computer vision-based fish detection. However, the performance of these YOLO models is limited due to adaptability to fish size, dynamic background, and computational time complexity. These were the significant challenges faced in most of the South Indian Pond environment. Hence it is necessary to enhance the existing YOLO models to make them adaptable for real-time aquaculture monitoring in South Indian ponds. This study introduces a novel research problem centered on detecting fish within pond environments specific to the Tamil Nadu region. To the best of our knowledge, this represents an unexplored area in the field, with minimal prior work dedicated to addressing this unique context. Earlier studies primarily utilized existing YOLO models, which faced limitations in addressing the challenges^40^ posed by unconstrained environments with diverse fish species and densely populated regions. To overcome these issues, AquaYOLO was specifically optimized for aquaculture applications. The model incorporates CSP layers and enhanced convolutional operations in the backbone to enable robust feature extraction. Its head integrates upsampling, concatenation, and multi-scale fusion for adaptive and effective feature representation. Additionally, the detection employs a precise 40$\times$40 scale and excludes the final C2f layer, ensuring accurate localization with reduced computational overhead. The key contributions of this work are:

- **New Application:** Introducing a novel and significant research problem centered on a real-time pond environment specific to the Tamil Nadu region.
- **Model Formation:** Developing “AquaYOLO” - a novel fish detection model that works in several real-time underwater challenges.
- **Model Validation and Comparison:** Validating AquaYOLO on our DePondFi dataset to evaluate its detection performance in real-time pond environments and comparing its performance with state-of-the-art models.
- **Model Generalization:** AquaYOLO has been evaluated on other fish detection benchmark datasets including DeepFish, OzFish, and DeepFish+OzFish dataset, achieving competitive performance.

The organization of this article is as follows: Section Materials and Methods details the methodology and details the AquaYOLO architecture, highlighting its key components and design principles. Section Experimental Design and Evaluation Framework provides a comprehensive performance evaluation of AquaYOLO, including a comparative analysis with state-of-the-art (SOTA) models on the DePondFi dataset. Furthermore, the model’s generalization capabilities are assessed using publicly available benchmark datasets. Section Results and Discussion concludes the article by summarizing the findings and outlining potential directions for future work.

## Materials and Methods

### AquaYOLO Model Architecture

**Figure 1:**
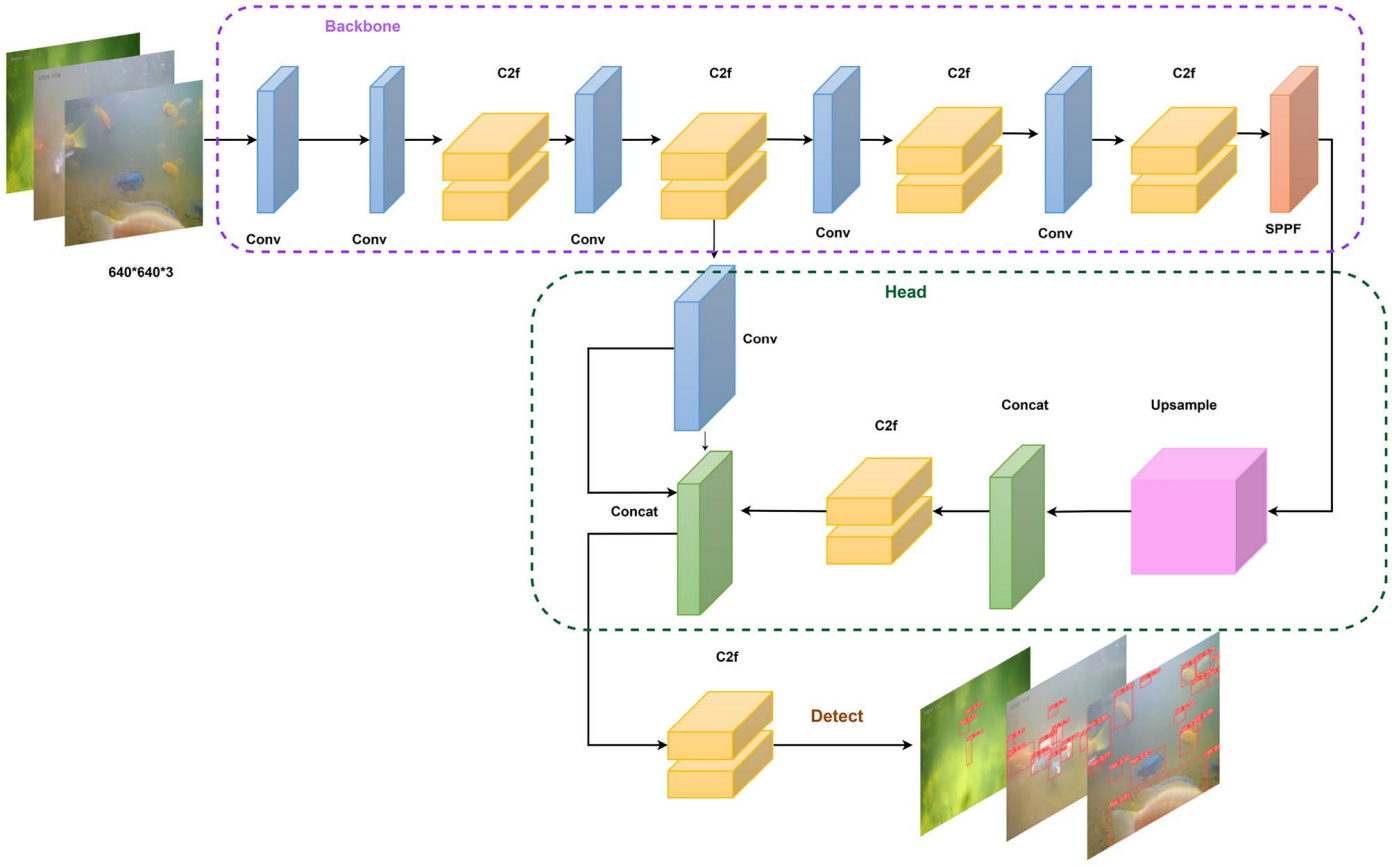
AquaYOLO architecture for fish detection.

AquaYOLO was designed to address the challenges of fish detection in complex underwater environments. AquaYOLO model architecture incorporates a Cross-Stage Partial (CSP) network and Spatial Pyramid Pooling Fusion (SPPF) to effectively generate feature maps of varying sizes through convolution and pooling operations. The objectness score within the output layer is activated through the sigmoid function for object presence and the softmax function for various object classes. The entire architecture of AquaYOLO is shown in Fig. 1 consists of three main parts backbone, head and detect.

#### Backbone:Feature extraction

The backbone is tasked with extracting detailed feature representations from the input images. It begins with an input layer that processes images of size $640 \times 640 \times 3$. The backbone includes multiple convolutional layers, each performing specific operations to transform the input data into feature maps of different resolutions. The first convolutional layer ($C_0$) processes the input image (*I*) with dimensions $(H_{\text {in}} \times W_{\text {in}} \times D_{\text {in}})$ using a kernel of size $k = 3$ ($K_0 \times K_0 \times D_{\text {in}} \times D_{\text {out}}$), where $b_0$ is the bias term, $p = 1$ is the padding, and $s = 1$ is the stride. The $*$ symbol denotes the convolution operation, resulting in an output ($P_1$) of size $320 \times 320 \times 6$. The convolution operation can be represented as:

1 $$\begin{aligned} C_0 = \text {Conv}(I, k=3, s=1, p=1) = I * K_0 + b_0 \end{aligned}$$

The output dimension is given by:

2 $$\begin{aligned} H_{\text {out}} = \frac{H_{\text {in}} - K_0 + 2p_0}{s_0} + 1, \quad W_{\text {out}} = \frac{W_{\text {in}} - K_0 + 2p_0}{s_0} + 1 \end{aligned}$$

where $p_0$ is the padding and $s_0$ is the stride.

Subsequent convolutional layers further downsample the feature maps. For instance, the second layer ($C_1$) applies a convolution with $k=3$, $s=2$, and $p=1$ to $C_0$, resulting in an output of size $160 \times 160 \times 128$.

3 $$\begin{aligned} C_1 = \text {Conv}(C_0, k=3, s=2, p=1) = C_0 * K_1 + b_1 \end{aligned}$$

The additional convolutional layers ($C_2$, $C_3$, and $C_4$) each reduce the spatial dimensions while increasing the depth of the feature maps, culminating in an output of size $20 \times 20 \times 1024$. The backbone also incorporates Cross-Stage Partial (CSP) layers, which split the feature maps into two parts, process them separately, and then merge them. This structure enhances gradient flow through the network. The CSP operation can be expressed as:

4 $$\begin{aligned} \text {CSP}_i = \text {Concat}(\text {Conv}(C_{i-1}), \text {Conv}(C_{i-1})) \end{aligned}$$

At the end of the backbone, the Spatial Pyramid Pooling Fast (SPPF)^41^ layer captures features at multiple scales by applying pooling operations with different kernel sizes and concatenating the results:

5 $$\begin{aligned} \text {SPPF} = \text {Concat}(\text {MaxPool}(C_{i-1}, k=5), \text {MaxPool}(C_{i-1}, k=9), \text {MaxPool}(C_{i-1}, k=13)) \end{aligned}$$

#### Head:Multi-Scale Feature Aggregation

The head aggregates features from different layers of the backbone to provide a multi-scale representation. It includes upsampling and concatenation layers to combine features from various resolutions. Initially, an upsampling layer ($U_0$) doubles the spatial dimensions of the SPPF output. This upsampled output is then concatenated with features from a previous layer ($CSP_0$).

The output from the SPPF layer $C_n$ is upsampled:

6 $$\begin{aligned} U_0 = \text {Upsample}(C_n, S_n) \end{aligned}$$

where $S_n$ is the scaling factor. The upsampled features are concatenated with features from $CSP_0$:

7 $$\begin{aligned} \text {Cat}_0 = \text {Concat}(U_0, \text {CSP}_0) \end{aligned}$$

The concatenated features are processed using the C2f block, which is represented as:

8 $$\begin{aligned} \text {C2f} = \text {Conv}(\text {Cat}_0, k_f, b_f) \end{aligned}$$

where $k_f$ and $b_f$ are the kernel size and bias for the convolution operation.

#### Detect:Bounding Box and Classification Prediction

The detection mechanism of AquaYOLO comprises detection layers responsible for predicting bounding boxes, objectness scores, and class probabilities. These detection layers operate on the multi-scale feature maps generated by the neck. The detection process applies a series of convolutional filters to generate predictions for each grid cell in the feature map. The final detection layer is represented as:

9 $$\begin{aligned} \text {Detect}_i = C_{\text {det}} * k_d + b_d \end{aligned}$$

where $C_{\text {det}}$ represents the input feature map, $k_d$ is the detection kernel, and $b_d$ is the detection bias.

#### Loss Function: Multi-Objective Optimization

The loss function combines localization, confidence, and classification losses:

10 $$\begin{aligned} L = \lambda _{\text {box}} L_{\text {box}} + \lambda _{\text {dfl}} L_{\text {dfl}} + \lambda _{\text {cls}} L_{\text {cls}} \end{aligned}$$

where $\lambda _{\text {box}}$ adjusts the importance of bounding box localization, $\lambda _{\text {dfl}}$ balances the contribution of objectness confidence, and $\lambda _{\text {cls}}$ weighs the influence of class classification. In the AquaYOLO architecture, the backbone begins with an input layer that processes images of size $640 \times 640 \times 3$. The input image is first passed through a convolutional layer, which applies a kernel size of $k=3$. This operation reduces the spatial dimensions of the image while increasing the depth, resulting in a feature map of size $320 \times 320 \times 64$. The next convolutional layer further downsamples this feature map using the same convolution parameters, producing a feature map of size $160 \times 160 \times 128$. Subsequent convolutional layers continue this process, progressively reducing the spatial dimensions while increasing the feature depth, culminating in a feature map with dimensions $20 \times 20 \times 1024$. The backbone also incorporates Cross-Stage Partial (CSP) blocks, which split the feature maps into two parts, process them separately through convolutional layers, and then merge them. This design enhances gradient flow and computational efficiency. At the end of the backbone, the Spatial Pyramid Pooling Fast (SPPF) layer captures features at multiple scales by applying pooling operations with kernel sizes of 5, 9, and 13. The head of AquaYOLO aggregates these multi-scale features to enhance detection accuracy. The SPPF output is upsampled to double its spatial dimensions, resulting in a feature map of size $40 \times 40 \times 512$. This upsampled feature map is concatenated with a feature map of matching dimensions from an earlier layer of the backbone. These concatenated features are then processed by a C2f block, which refines the feature representations. Finally, the detection layers predict bounding boxes, objectness scores, and class probabilities. These layers operate on feature maps of different scales ($80 \times 80$, $40 \times 40$, and $20 \times 20$) to ensure multi-scale detection. The outputs include bounding box coordinates, object confidence scores, and class probabilities, enabling accurate fish detection across varying object sizes and locations. AquaYOLO focuses on the $40 \times 40$ scale to optimize by balancing fine spatial details with high-level semantic features. The architecture employs efficient feature fusion in the head, integrating shallow spatial features with deep semantic representations, ensuring accurate object localization. The combination of convolutional layers, CSP layers, and SPPF in the backbone, along with multi-scale feature aggregation and precise detection capabilities in the head, ensures robust and accurate object detection. This architecture is further modified with layers C2f and pooling features at varying scales, resulting in three variants: AquaYOLO#1, AquaYOLO#2, and AquaYOLO#3. Among these, the best-performing model for the DePondFi dataset^42^ is selected, and hyperparameter tuning is performed to achieve optimal performance. The AquaYOLO architecture has a parameter size of 53.1M and a model size of 106.6 MB. The AquaYOLO algorithm for fish detection is elucidated in Algorithm 1. The algorithm describes the fish detection process in AquaYOLO, utilizing Non-Maximum Suppression (NMS) to refine detection outputs. It iteratively selects the detection box with the highest confidence score and includes it in the final set of results. Subsequently, overlapping boxes are removed based on the Intersection over Union (IoU) and the specified NMS threshold, ensuring that the final detections are both accurate and non-redundant.

**Algorithm 1:**
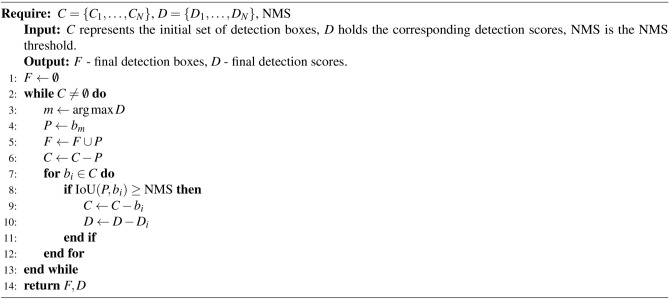
Fish Detection Technique using AquaYOLO

## Experimental design and evaluation framework

To implement fish detection in wild underwater environments, model training was performed using the Anaconda platform and Google Colab Pro. The training was performed on Colab Pro, utilizing a Tesla 4 GPU and 25 GB of RAM. Models were tested using PyTorch in a conda environment on a NVIDIA GEFORCE GTX 1650 GPU system. The system used for these tests was configured with an AMD Ryzen 5 5600H processor operating at 3.30 GHz, 8 GB of graphics RAM, and a 64-bit operating system based on an x64 architecture. The primary evaluation metrics included recall, precision, and mean Average Precision (mAP). Specifically, the mean Average Precision at an Intersection over Union (IoU) threshold of 0.95 (mAP@95), which is analogous to mAP@50, was used to measure the performance of object detection models under stricter IoU requirements. A comprehensive evaluation of the models was conducted by analyzing these key metrics, including recall, precision, and mAP.

## Results and discussion

### DePondFi dataset details

The DePondFi [42] dataset was curated by collecting real-time videos from aquaculture farms using underwater cameras. The study area includes various aquaculture farms in Tamil Nadu: Cuddalore, Kanchipuram, Chennai, and Tanjore. These districts represent diverse pond environments, each with unique characteristics relevant to aquaculture.

**Pond-1 Series (Cuddalore):** This series includes villages such as Thukkanampakkam, Thenampakkam, and Pallipattu, covering a total area of 6 acres across 10 ponds. Each pond measures approximately 10,000 square meters with a depth of 4 meters, characterized by high turbidity levels. Common species cultivated include Carp, Rogu, Catla, Mrigal, Silver Carp, Roopchanda, Tilapia, and Grass Carp.

**Pond-2 Series (Tanjore):** Located in Tanjore, the Pond-2 series consists of 19 ponds, each dedicated to different fish species such as Catla, Common Carp, Mrigal, Rohu, Silver Carp, and Grass Carp. These ponds are characterized by mixed species cultivation and specific male and female fish ponds. Videos were captured at intervals in the morning and evening, at a resolution of 720p at 60fps.

**Pond-3 Series (Kancheepuram):** The ponds in Kancheepuram are surrounded by cultivated lands and have lower turbidity levels due to the absence of artificial fertilizers. Species such as Catla, Rogu, and Mrigal are cultivated here. Videos were captured in two settings, 720p at 60fps and 1080p at 60fps, reflecting the organic farming practices in the region.

**Pond-4 Series (Chennai):** Situated in Kolathur, Chennai, Pond Series 4 focused on fast-growing species like Catla, Rohu, Tilapia, Catfish, and Pangasius. This area includes over 1,000 fish farms, also emphasizing ornamental fish production. Videos were recorded at 720p and 1080p at 60fps for 30 minutes each. The details of Pond series visited for the dataset collection is described is tabulated in Table 1.

**Table 1:** Details of the study area.

Series	Area (acres)	Depth (m)	# Fish Varieties	# Frames
Pond 1	6	1	8	2200
Pond 2	19	4.5 – 5	13	2150
Pond 3	1	2 – 3	6	1200
Pond 4	2	1 – 2	20	2600

**Figure 2:**
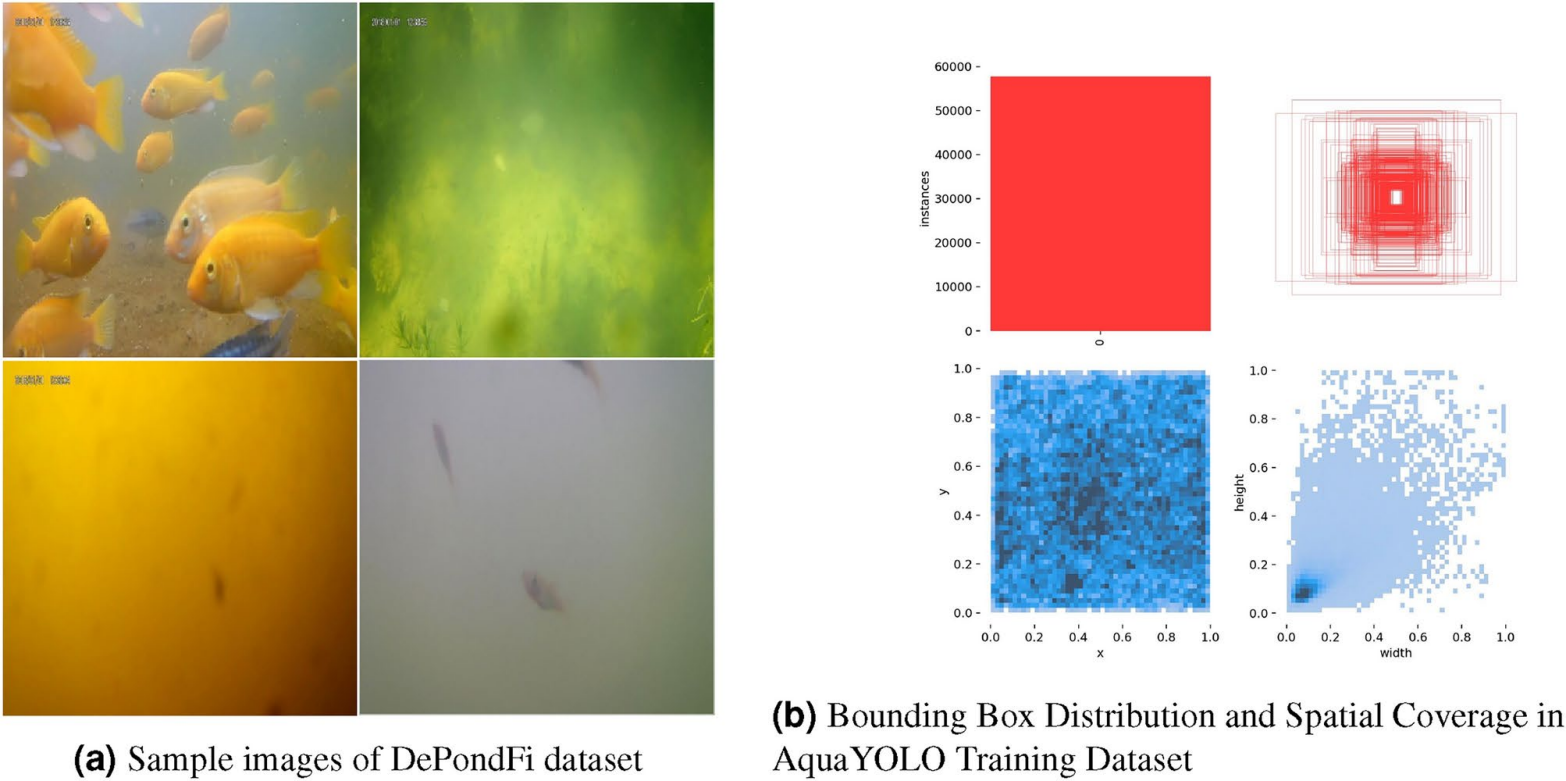
Combined visualization of the DePondFi dataset and AquaYOLO bounding box distribution.

The video data is manually processed through keyframe extraction to isolate relevant frames, which are subsequently resized to $640 \times 640$ dimensions. The extracted frames are annotated using Roboflow software, employing a bounding box annotation format to precisely define object regions for detection.

### AquaYOLO model performance analysis

DePondFi dataset used for model evaluation was divided into training, validation, and testing sets in proportions of 70%, 20%, and 10%, respectively, to facilitate accurate model assessment.There were 5705, 1630, and 815 labeled images in the train, validation, and test sets, respectively. The model training was carried out with a well-defined set of parameters to achieve effective performance. Training ran for 25 epochs with early stopping applied after 15 epochs if no improvement was observed. A batch size of 64 was used with two data loaders to handle the dataset. The initial and final learning rates (lr0 and lrf) were both set to 0.01. Stochastic Gradient Descent (SGD)^43^ was used as the optimizer, configured with a momentum of 0.95 and a weight decay of 0.0001. A 10-epoch warm-up was included, where the momentum was set to 0.5, and the bias learning rate was adjusted to 0.1. Pretrained weights (aquayolo1.pt, aquayolo2.pt, and aquayolo3.pt) were utilized, and input images were resized to 640 × 640 pixels. The annotations were prepared in YOLOv8 format (TXT files) with a bounding box style. The backbone architecture for the model was CSPDarknet53, and the dataset included around 50,000 instances. These settings ensured stable training and good results. The sample images from our DePondFi dataset are shown in Fig. 2a. Figure  2b illustrates the distribution and characteristics of the AquaYOLO training dataset. The top-left plot shows the distribution of class instances, highlighting a balanced dataset with no significant class imbalances. The top-right plot overlays all bounding boxes, revealing a dense central region where most objects are concentrated, typical in controlled environments like aquaculture ponds. The bottom-left plot visualizes the spatial heatmap of object locations, indicating a relatively uniform distribution across the image frame. The bottom-right plot displays the width and height ratios of bounding boxes, with a dense cluster in the lower-left corner suggesting the presence of numerous small-scale objects.

Our proposed AquaYOLO model is evaluated on the DePondFi dataset, along with its variants. The quantitative results, presented in Table 2, demonstrate that the proposed AquaYOLO model outperforms its variants, achieving the highest detection accuracy with a mAP@50 of 0.909 and an mAP@50-95 of 0.520. Additionally, AquaYOLO exhibits superior computational efficiency, requiring only 1.38 hours for training, with minimal pre-processing (0.1 ms) and post-processing (1.1 ms) times. These results establish AquaYOLO as an efficient solution for real-time aquaculture monitoring applications.

**Figure 3:**
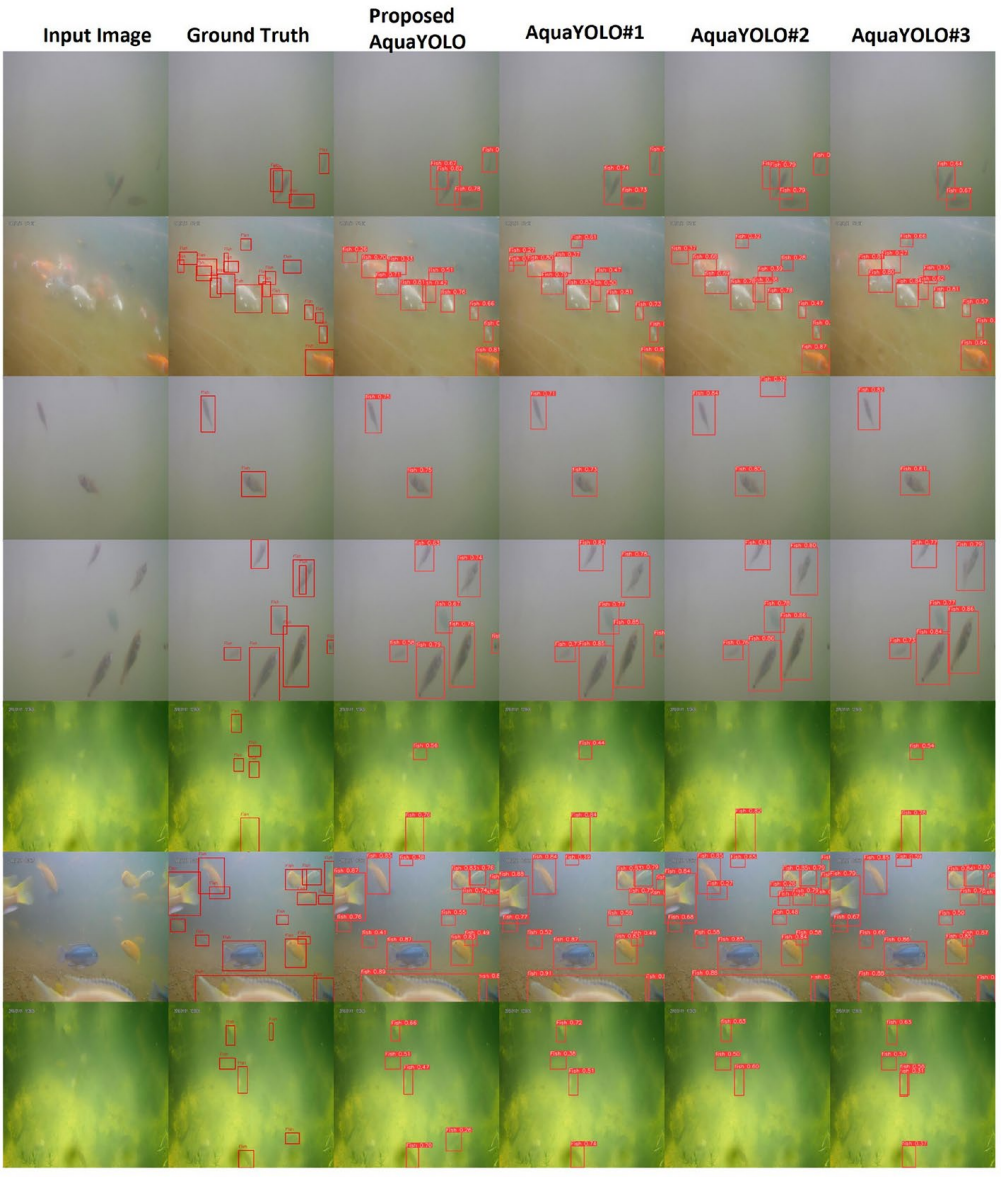
Qualitative results of our proposed models on DePondFi.

Figure  3 illustrates the qualitative results of the proposed AquaYOLO model and its variants on the DePondFi dataset, evaluated under diverse underwater environmental conditions. The first column presents the input images, showcasing varying levels of visibility, object density, and turbidity. The second column displays the manually annotated ground truth bounding boxes, providing a reference for evaluating the detection accuracy of the algorithms. The subsequent columns compare the detection outputs of the proposed AquaYOLO model and its variants (AquaYOLO#1, AquaYOLO#2, and AquaYOLO#3). The proposed AquaYOLO model demonstrates robust detection performance, accurately identifying fish with high confidence scores (e.g., ‘Fish 0.85,’ ‘Fish 0.92’), even in challenging scenarios with overlapping objects and low visibility. In contrast, AquaYOLO#1 struggles with complex backgrounds, often leading to missed detections. AquaYOLO#2 and AquaYOLO#3 exhibit variable detection accuracy, particularly for smaller fish, with occasional false positives or lower confidence scores. The proposed AquaYOLO model consistently aligns closely with the ground truth annotations, outperforming its variants in terms of both accuracy and reliability.

**Table 2:** Quantitative performance comparison of AquaYOLO variants on the DePondFi dataset.

MODELS	Image Size	Epoch	mAP@50	mAP@50-95	GFLOP	Parameter	IoU	Prediction-Time(ms)
AquaYOLO#1	640$\times$640	25	0.881	0.474	119.3	16,280,800	0.5	0.2
AquaYOLO#2	640$\times$640	25	0.893	0.497	150.5	53,187,552	0.5	0.2
AquaYOLO#3	640$\times$640	25	0.874	0.489	134.3	80,158,688	0.5	0.3
Proposed AquaYOLO	640$\times$640	25	0.909	0.520	150.3	53,147,025	0.5	0.1

Our proposed AquaYOLO model was evaluated against state-of-the-art (SOTA) detection models under consistent hyperparameter settings to ensure uniform evaluation. Here the YOLO version ($x$) and SOTA models are taken for the comparison. The performance metrics, including precision, recall, mAP@50, and mAP@95, are summarized in Table 3. AquaYOLO achieved a precision of 0.889, recall of 0.848, mAP@50 of 0.909, and mAP@95 of 0.52, outperforming the majority of the SOTA models and demonstrating its effectiveness in challenging aquaculture environments. The number of epochs was set to 25 to prevent overfitting, as increasing the epochs led to a decline in model generalization.

**Table 3:** Quantitative performance comparison of the proposed AquaYOLO model with state-of-the-art (SOTA) detection models.

MODEL	Precision	Recall	mAP@50	mAP@95	Parameters	Backbone
Faster R-CNN	0.853	0.841	0.878	0.472	19M	ResNet-50-FPN
EfficientDet	0.847	0.827	0.890	0.491	52M	EfficientNet(D7)
RetinaNet	0.834	0.832	0.879	0.454	32M	ResNet-50
SSDv2	0.862	0.823	0.868	0.452	15M	MobileNet
YOLOv5	0.848	0.799	0.856	0.424	46.5M	CSPDarknet53
YOLOv7	0.892	0.854	0.912	0.496	75.6M	ELAN
YOLOv8	0.885	0.842	0.905	0.510	68.2M	CSPDarknet53
YOLOS	0.757	0.694	0.760	0.273	9M	Vision Transformer
Proposed AquaYOLO	0.889	0.848	0.909	0.520	53.1M	Modified CSPDarknet53

### AquaYOLO model evaluation on DeepFish, OzFish and Deep+OzFish

To evaluate the generalization capability of the proposed AquaYOLO model, its performance was assessed on publicly available benchmark datasets, namely DeepFish and OzFish. The DeepFish dataset contains 4,505 images with bounding box annotations, while the OzFish dataset comprises 2,305 annotated images. Furthermore, a cross-dataset evaluation was conducted by combining and augmenting both datasets, resulting in a unified dataset of 7,763 images. The AquaYOLO model was systematically evaluated across these datasets to validate its generalization performance.

**Figure 4:**
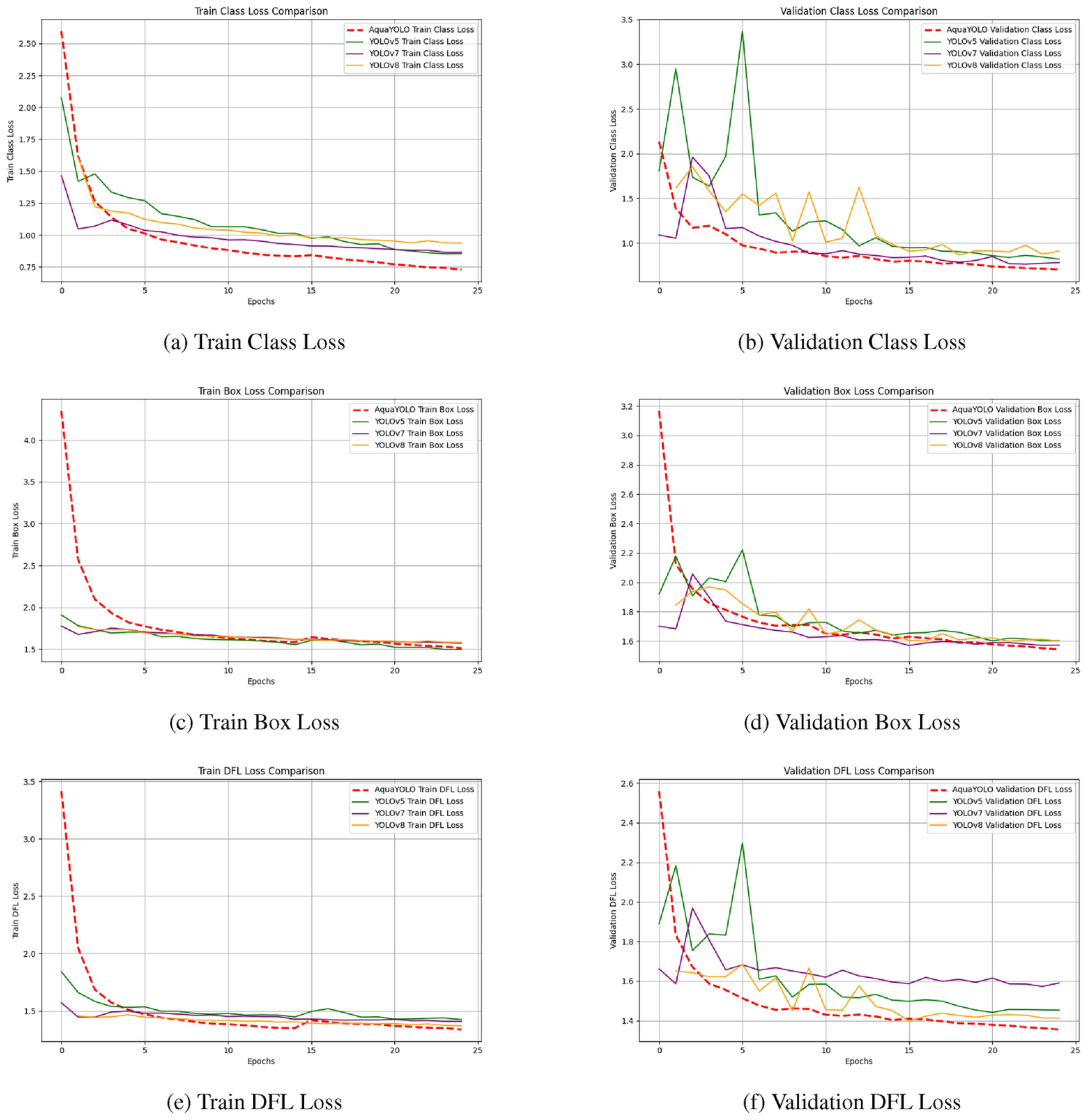
Combined loss curves over 25 epoch for SOTA - Top 4 models.

Table  4 summarizes the performance of AquaYOLO on three datasets. The combined dataset achieves the best results with a precision of 0.885, recall of 0.857, mAP@50 of 0.814, and mAP@50-95 of 0.473. The train and validation class loss comparison shown in Fig.  4 depicts the evolution of class prediction errors during training and validation phases. AquaYOLO demonstrates a consistent reduction in class loss compared to other models, achieving convergence at lower loss values. The train and validation box loss comparison illustrate the ability of the models to predict accurate bounding box coordinates. AquaYOLO shows a steady decrease in box loss, converging faster and more efficiently than its counterparts, especially under challenging conditions. The train and validation dfl (Distribution Focal Loss) loss comparison evaluates the models’ ability to optimize fine-grained localization and confidence scores.

**Table 4:** Quantitative comparison of AquaYOLO on benchmark datasets.

Benchmark Dataset	Precision	Recall	mAP@50	mAP@50-95
DeepFish^25^	0.618	0.668	0.420	0.453
OzFish^24^	0.774	0.776	0.591	0.424
DeepFish+OzFish	0.885	0.857	0.814	0.473

**Figure 5:**
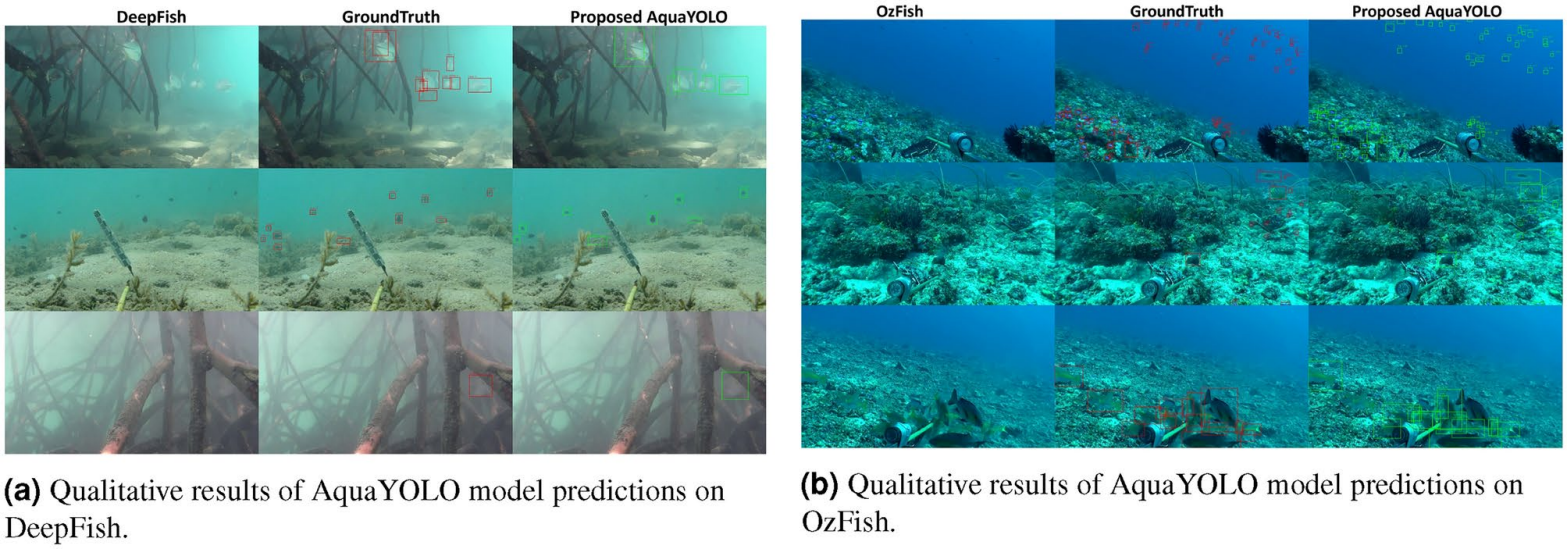
Qualitative results of AquaYOLO model predictions on two datasets: DeepFish and OzFish.

Figure  5a presents the qualitative results of the AquaYOLO model on the benchmark datasets DeepFish^25^ and OzFish^24^ is shown in Fig.  5b, evaluated under diverse underwater scenarios. In the DeepFish dataset, AquaYOLO demonstrates its robustness in detecting fish within cluttered underwater environments. Despite the presence of overlapping objects and complex vegetation, the model accurately localizes fish, as indicated by the green bounding boxes. The alignment of predictions with ground truth (red boxes) shows AquaYOLO’s high precision and reliability, even under challenging conditions. In the OzFish data set, AquaYOLO effectively handles varying fish sizes, sparse distributions, and uneven lighting. The green bounding boxes illustrate the model’s ability to detect fish across a range of environmental settings. The results highlight the superior generalization performance of AquaYOLO, particularly in clear water conditions where the fish are distributed at different depths.

### Evaluation of existing model on DePondFi dataset

Table 5 compares the performance of models tested on the DePondFi dataset. Models such as YOLOFish (mAP: 0.1569) and Detectron (mAP: 0.389) demonstrate significant limitations in accuracy and computational efficiency. YOLOFish, with a relatively low mAP and a processing time of 0.87 seconds per image, struggles to handle the complexities of the dataset. Detectron performs moderately better in terms of mAP but still requires 0.036 seconds per image, indicating room for improvement in efficiency. These results highlight the challenges posed by the DePondFi dataset and the need for more effective solutions for underwater detection tasks. The proposed AquaYOLO model outperforms other methods with an mAP of 0.52, addressing the challenges of the DePondFi dataset.

**Table 5:** Comparison of the performance of existing models evaluated on the DePondFi dataset.

Model Name	mAP	Preprocessing	Time (sec)
YOLOFish^13^	0.1569	Normalized	0.87
Detectron^42^	0.389	Normalized	0.036
DMACS_SAI^42^	0.3665	-	0.001
PondVision^42^	0.3163	-	0.006
Sahajeevis^42^	0.291	-	0.001
Ours (AquaYOLO)	0.52	-	0.0001

### Limitation and future work

The AquaYOLO model has demonstrated robust performance in underwater environments; however, it faces limitations under specific conditions. Detecting small or distant fish remains challenging, particularly when fish are far from the camera or in regions with extreme turbidity, where visibility is severely compromised. Additionally, the model exhibits difficulties in detecting fish obscured by significant occlusions, such as overlaps involving more than five fish. Despite these challenges, AquaYOLO supports essential aquaculture processes, including fish counting, species identification, and biomass estimation. The integration of AquaYOLO with advanced sensors can enable real-time water quality monitoring and environmental change detection, ensuring optimal conditions for fish health. Moreover, the model’s ability to provide accurate real-time results makes it highly suitable for integration with automated feeding systems, minimizing feed waste and enhancing resource utilization. Future work will focus on addressing the current limitations by incorporating advanced techniques for handling occlusions, improving detection of small and distant objects, and optimizing the model for low-visibility conditions to further enhance its applicability in diverse aquaculture scenarios.

## Conclusion

In this study, we developed and validated AquaYOLO, a fish detection model designed for real-time aquaculture monitoring in South India. The model addresses challenges such as variable lighting, water depth, and turbidity, demonstrating significant improvements in efficiency and accuracy compared to existing methods. Key architectural enhancements, including optimized C2f and convolutional layers, enabled AquaYOLO to achieve a precision of 0.889, a recall of 0.848, and a mAP@50 of 0.909. The model’s performance was evaluated against state-of-the-art models, showing superior detection capabilities. AquaYOLO was also validated on publicly available datasets to confirm its generalization ability. This work represents an important advancement in aquaculture monitoring, improving detection accuracy and operational efficiency. By supporting Sustainable Development Goal 14 (Life Below Water), AquaYOLO contributes to the sustainable management of aquatic resources, aiding in the preservation of marine ecosystems and supporting global food security initiatives.

## Data Availability

All data generated or analysed during this study are included in this published article.
